# Physical activity during pregnancy and the risk of gestational diabetes mellitus: a systematic review and dose–response meta-analysis

**DOI:** 10.1186/s12889-024-18131-7

**Published:** 2024-02-23

**Authors:** Wanting Xie, Liuwei Zhang, Jiaoying Cheng, Yirui Wang, Haixin Kang, Yi Gao

**Affiliations:** 1 Department of Physical Fitness and Health, School of Sport Science, Beijing Sport University, Beijing, 100084 China; 2 Key Laboratory of Exercise and Physical Fitness, Ministry of Education, Beijing Sport University, Beijing, 100084 China; 3 Department of Obstetrics and Gynecology, China-Japan Friendship Hospital, Beijing, 100029 China; 4 School of Strength and Conditioning Training, Beijing Sport University, Beijing, 100084 China

**Keywords:** Physical activity, Gestational diabetes mellitus, Systematic review, Dose–response, Meta-analysis

## Abstract

**Background:**

Previous research has indicated the inverse association between physical activity (PA) and gestational diabetes mellitus (GDM). However, the dose–response relationship currently remains undetermined. This study aims to explore the dose–response relationship between PA during the first and second trimesters of pregnancy and GDM risk.

**Methods:**

Studies on the relationship between PA during pregnancy and GDM risk published before April 25, 2023, were searched for in six databases. According to the inclusion and exclusion criteria, all literature was screened for eligibility. The Newcastle-Ottawa Scale (NOS) was used to assess risk of bias. Publication bias was examined using funnel plots, Begg’s and Egger’s tests, as well as trim-and-fill analysis. We harmonized exposure estimates of PA during pregnancy to the common unit of the metabolic equivalent of task (MET)-h/week. Restricted cubic splines were used to model the dose–response relationship. The criteria from the World Cancer Research Fund were used to assess the certainty of evidence across outcomes. All analyses were performed using Stata 15.1.

**Results:**

The results indicated that in contrast with the lowest level of PA, promoting the highest PA level lowers the risk of GDM by 36% (RR = 0.64, 95%CI: 0.53 ~ 0.78). We found a curvilinear dose–response association between PA during the first trimester and incident GDM (*P*_nonlinearity_ = 0.012). Compared to inactive pregnant women, for those who achieved the guidelines-suggested minimum level (10 MET-h/week) of PA during the first trimester, the GDM risk was decreased by 13% (RR = 0.87, 95%CI: 0.79 ~ 0.96). A linear relationship was found between PA during the second trimester and the GDM risk (*P*_nonlinearity_ = 0.276). The results with a restricted cubic spline model suggested that pregnant women who accumulate 10 MET-h/week have a 1% reduced risk of GDM compared to completely inactive individuals. Twice (20 MET-h/week) or a higher amount of PA (50 MET-h/week) contributed to further reductions in GDM risk.

**Conclusion:**

There is a dose–response relationship between higher levels of PA in both the first and second trimesters and reduced risk of GDM; the relationship is stronger in the first trimester. Increasing PA during pregnancy can prevent the development of GDM.

**PROSPERO registration number:**

CRD42023420564.

**Supplementary Information:**

The online version contains supplementary material available at 10.1186/s12889-024-18131-7.

## Introduction

Gestational diabetes mellitus (GDM) is one of the most prevalent pregnancy complications. Since the World Health Organization (WHO) identified GDM as an individual type of diabetes in 1997, GDM has attracted considerable attention in the fields of obstetrics and gynaecology [1, 2]. With the evolution of civilization and a subsequent improvement in living standards, the prevalence of global GDM has displayed a significant upward trend, impacting over than 20 million women during pregnancy by 2019 [3, 4]. The health of both the mother and infant is adversely affected by GDM: pregnant women with GDM have a greater risk of obesity and hypertension in the short term, while the long-term risks for offspring born to mothers with GDM include an increased risk of developing type 2 diabetes mellitus (T2DM) [5, 6]. Thus, strengthening efforts to prevent GDM is critical.

Various genetic and environmental factors are known to be related to the risk of developing GDM [7]. Research has indicated that physical activity (PA) plays a role in improving insulin sensitivity, directly or indirectly, through a variety of mechanisms that influence the risk of developing diabetes [8]. Compared to genetics, a low level of PA during pregnancy is a potentially modifiable factor with more practical significance for the prevention and management of GDM. Low PA during pregnancy is an individual behavioural risk factor for GDM and leads to an increased number of risks associated with pregnancy complications [9–11]. According to the 2015 Opinion of the American College of Obstetricians and Gynaecologists Committee (ACOG), ‘Physical activity in pregnancy has minimal risks and has been shown to benefit most women’. While this is intended to encourage women to increase their PA levels during pregnancy as a means to reduce their risk of developing GDM [12, 13], it should be noted that excessive PA can put pregnant women at risk for additional hazards, such as muscle damage and even foetal death [14].

A previous review has indicated a curvilinear relationship between PA and incident T2DM [15]. Given that GDM and T2DM share a similar pathophysiology, it makes sense to believe that a similar dose–response relationship exists between PA and GDM; this could provide an appropriate recommendation of PA levels to reduce GDM risk for pregnant women. Unfortunately, the dose–response relationship between PA during pregnancy and GDM risk has not been systematically evaluated. First, previous studies only investigated the association between specific aspects of PA and the GDM risk. The majority of recent studies on PA during pregnancy have focused on the association between GDM and the type, intensity and duration of the PA as well as the ethnicity of the pregnant woman. Feng et al. found that various types of PA during the first trimester reduced the risk of GDM in different ways [16]; for example, household/caregiving and sports/exercise during pregnancy were discovered to be effective in lowering GDM risk when compared to transportation and occupational activities. Meanwhile, it has been demonstrated that varying the intensity and duration of PA may contribute to different GDM risks. Previous research has demonstrated that moderate-to-vigorous PA (MVPA) during pregnancy reduces the risk of GDM more than lower-level PA for the same duration but at a different intensity [17]. In 2016, a study found that compared to women with a weekly leisure-time PA duration of less than 3 hours during their first trimester, women who participated in weekly leisure-time PA for over 6 hours had a 18% reduced risk of developing GDM [18]. Additionally, women from Asia have been reported to have a higher GDM risk compared with the majority of the global population. According to the results of a study that documented PA and its association with GDM, South Asian women who had the same levels of MVPA during pregnancy as women in Western Europe nevertheless had a 92% increased risk of GDM [19]. Second, the distinct definitions and classification of PA levels during pregnancy utilised by different studies have led to the development of different PA units, thereby making it difficult to systematically assess the dose-response relationship between PA during pregnancy and the GDM risk. Third, it is necessary to promptly update previous evaluations of the dose–response relationship between PA during pregnancy and the risk of GDM as well as examined this relationship in the second trimester. The non-linear relationship between PA before and during the first trimester of pregnancy and the risk of developing GDM was reported in a meta-analysis published in 2016 [20]. However, the meta-analysis included fewer studies due to its early publication date. Additionally, Aune et al. focused solely on the dose–response relationship between PA before and during the first trimester of pregnancy, thereby overlooking its impact on GDM risk in the critical second trimester. Furthermore, additional PA-related data from the Chinese populations that were not previously considered are essential for analysing the dose–response relationship.

Consequently, to provide a more reliable theoretical basis for the prevention of GDM, our study intends to include the latest evidence, including data on the Chinese population, to systematically evaluate the dose-response relationship between PA during the first and second trimesters of pregnancy and risk of developing GDM in order to provide more suitable exercise suggestions for preventing GDM in gravid women.

## Materials and methods

All meta-analyses performed in this paper strictly followed the Preferred Reporting Items for Systematic Reviews and Meta-Analyses (PRISMA) statement [21].

The registration number for this meta-analysis on PROSPERO is CRD42023420564 (available from https://www.crd.york.ac.uk/prospero/display_record.php?ID=CRD42023420564).

### Literature search

The electronic databases CNKI, Wanfang, VIP, Web of Science, PubMed, and those in the EBSCO series were searched for all literature on PA during pregnancy and the risk of developing GDM; the following keywords were searched for using the corresponding search formula (see Table S1 in the Supplementary Information): ‘pregnancy/pregnant women/maternal/gestation’, ‘gestational diabetes mellitus/blood glucose’, and ‘exercise/physical activity/physical fitness/sport/lifestyle intervention/exercise intervention’. The search period is from the establishment of the database to April 25, 2023. The Language was limited to Chinese and English.

### Inclusion and exclusion criteria

Studies were included if they (1) were classified as either cohort or case-control; (2) excluded gravida with GDM at baseline in cohort studies or a no-GDM population as a control group in case-control studies; (3) ascertained levels of PA at baseline; (4) used validated PA instruments to estimate PA levels during pregnancy; (5) involved GDM outcomes; (6) reported relative risks (RRs), odds ratios (ORs) with 95% confidence intervals (CIs) or supplied data that enabled calculation.

Studies were excluded if they were (1) those studies classified as cross-sectional or randomised controlled trial studies; (2) cohort studies in which the subjects were GDM participants at baseline; (3) studies that reported only the association between pre-pregnancy PA or continuous data and GDM risk; (4) those which provided no details or provided insufficient information on PA assessment to estimate doses in terms of metabolic equivalent of task (MET)-h/week.

Two researchers (W.X. & L.Z.) conducted the literature search and screening process independently; they screened titles and abstracts for eligibility according to the inclusion and exclusion criteria. Full texts were retrieved in cases where eligibility was ambiguous. Any discrepancies were resolved through discussion.

### Data extraction and exposure harmonisation

Using Excel, data were extracted on first author, year of publication, country, race, age, population, type of study, gestational trimester, GDM diagnostic criteria, method of PA during pregnancy assessment, reported levels of PA during pregnancy, total population and cases of GDM per category of PA, RRs, or ORs for GDM with 95% CIs for each PA category.

Prior to the primary analysis, estimates of PA during pregnancy reported in each study were harmonised to calculate a dose that could be used for analysis in the study. We initially harmonised group-level exposure estimates to the common unit of MET-h/week, thereby making it usable for the integration of activities accumulated throughout a week, which have a range in terms of intensity and duration.

To categorise PA with specific intensities, light PA, moderate PA, MVPA and vigorous PA were given mean intensities of 3, 4, 4.5, and 8 METs, respectively [22]. When the PA volume was not directly reported, the median or midpoint duration of the reported category was multiplied by the MET value that was assigned to determine the PA volume (MET-h/week). In addition, the interval width was presumed to be the same that in the closest category if the highest category for PA duration was open-ended. Zero was selected as the lower boundary when the lowest category was left unspecified. Further, for instances in which the PA intensity was unidentified, we assumed that it was 4.5 METs. In the main analysis, a single session was assumed to last 45 minutes if PA was reported only as the frequency of sessions per week, and a 30-minute assumption was utilised in the sensitivity analysis to determine the stability of the results.

Additionally, studies with reported ORs for GDM were judged to be approximately equivalent to RRs. Articles that reported data separately for the first and second trimesters were treated as independent studies. Further, studies that reported risk estimates based on the highest PA category underwent recalculation using the lowest PA group as the referent.

### Quality assessment

Reviewers used the Newcastle-Ottawa Scale (NOS) to assess the quality of the included studies [23]. The scale was divided into three sections, with a total score of nine. Studies with scores from six to nine were considered to be of a high quality and sufficient for inclusion in the meta-analysis. Any disagreements arising between quality evaluations were settled by the third author (J.C.).

### Statistical analysis

Based on the results of the heterogeneity test, either a fixed-effects model or a random-effects model was selected for the combined study-specific RR estimates and 95% CIs for the highest versus the lowest level of PA. *I*^*2*^ statistics evaluated heterogeneity. *I*^*2*^ values above 50% for the *I*^*2*^ statistic were regarded as reflecting high heterogeneity [24]; therefore, subgroup analyses were performed on race. If the population in a study included more than one race, that population was analysed as a multi-ethnic subgroup. Moreover, sensitivity analysis was used to test the stability of the results, and publication bias was estimated using a funnel plot, trim-and-fill analysis and Egger’s and Begg’s tests, with a significance threshold of *P < 0.05*.

Generalised least squares regression was used to estimate study-specific dose–response association [25]. First, assuming a linear relationship between PA and risk of GDM, RR values were calculated for each increment of 10 MET-h/week (equivalent to 150 min/week at the minimum guideline-recommended level of moderate physical activity during pregnancy [12]), 20 MET-h/week (double the minimum level of PA recommended), and 50 MET-h/week to calculate the risk of GDM associated with an increase of 10 MET-h/week, 20 MET-h/week, and 50 MET-h/week during pregnancy. Additional exploration of any non-linear relationships was performed by modelling with a restricted cubic spline and three knots placed at the 25th, 50th, and 75th percentiles of the distribution. Only studies reporting risk estimates for at least 3 PA exposure levels for incident GDM were included in the dose–response analysis. The *P*-value for non-linearity was determined by testing the null hypothesis that the coefficient of the second spline was equal to zero [26]. All analyses were performed using Stata 15.1 software.

### Certainty of the evidence

In this meta-analysis, we assessed the certainty of the evidence for outcomes in accordance with the criteria provided by the World Cancer Research Fund [27], which can be considered an assessment of the risk of non-communicable diseases related to PA. These criteria led to five possible levels of conclusion: convincingly causal, probably causal, limited evidence, no conclusion of a causal relationship possible, and substantial effect on risk unlikely.

## Results

### Literature screening included studies’ characteristics and NOS score

**Fig. 1:**
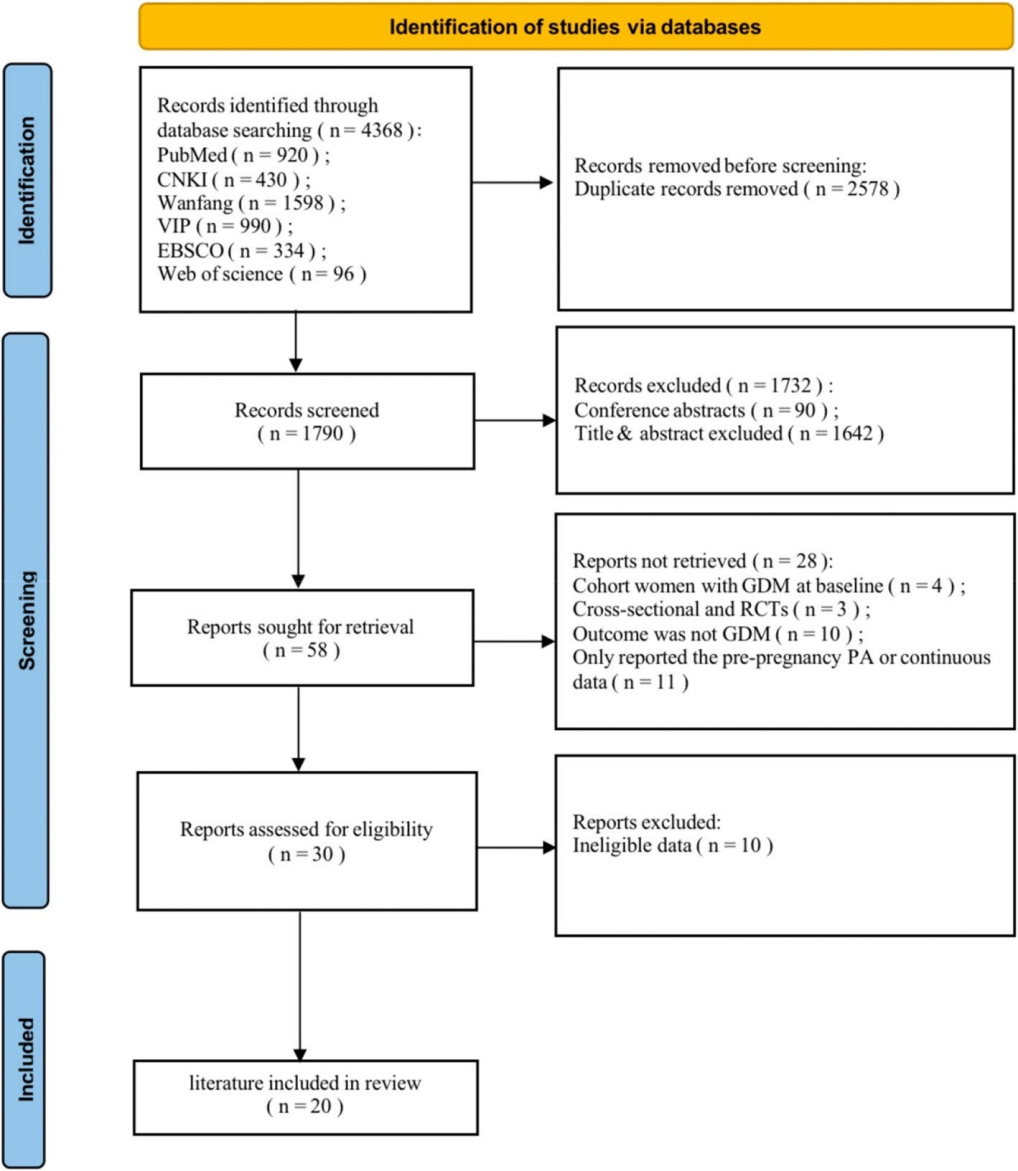
Flowchart of Study Selection for the Meta-analysis

The initial search yielded 4368 relevant articles in the database. Following the elimination of 4348 ineligible articles, 20 articles were ultimately included (a total of 22 independent studies), which included 40,485 pregnant women and 4402 GDM participants [10, 14, 16–18, 28–42]. Seven studies were conducted in America, six in China, five in other Asian countries, one in Ethiopia, and one in Brazil. Except for two studies in China and America, all included studies provided GDM criteria (five studies used the International Association of the Diabetes and Pregnancy Study Groups, four used the American Diabetes Association, three used the National Diabetes Data Group, three used the World Health Organization’s criteria, two used Carpenter and Coustan criteria, and one adhered to the Guideline of Gestational Diabetes Mellitus, 2014). Furthermore, all articles used validated PA instruments or interviews to measure PA levels during pregnancy. Of the 22 independent studies, 11 estimated PA in the first trimester and 11 assessed PA in the second trimester. All the included studies had NOS quality scores of six or higher, thereby indicating high study quality of the study. The specific screening procedure is depicted in Fig. 1. The basic characteristics, calculated PA dose, and the results of the quality assessment of the included literature are presented in Table 1; detailed information on the included studies according to the PECOS is presented in Table S2 in the Supplementary Information.

**Table 1:** The characteristics of all qualified studies in meta-analysis

No.	Author	Year	Country	Race	Population	Type	Gestational trimester	Criteria of GDM	PA assessment (dose in MET-h/week)	RRs/ORs (95%CIs)	NOS
1	Feng et al. [16]	2020	China	Asian	909	Cohort	First trimester	IADPSG	low (53.79)	1.00	8
									Moderate (164.74)	0.922 (0.625 ~ 1.361)	
									High (279.06)	0.970 (0.659 ~ 1.428)	
2	Hu et al. [14]	2021	China	Asian	669	Case-control	Second trimester	IADPSG	Q1 (43.45)	1.00	7
									Q2 (102.503)	1.660 (0.956 ~ 2.884)	
									Q3 (137.40)	0.759 (0.416 ~ 1.384)	
									Q4 (175.949)	0.386 (0.200 ~ 0.743)	
3	Xie et al. [28]	2016	China	Asian	6211	Case-control	Second trimester	NA	< 7 h/week (10.5)	1.00	7
									7 ~ 10 h/week (25.5)	1.109 (0.928 ~ 1.324)	
									> 10 h/week (34.5)	0.980 (0.830 ~ 1.174)	
4	Ma [29]	2019	China	Asian	3646	Cohort	First trimester	IADPSG	0 times /week (0)	1.00	8
									1 ~ 7 times/week (18)	0.90 (0.59 ~ 1.38)	
5	Wu et al. [30]	2020	China	Asian	1083	Cohort	Second trimester	IADPSG	Q1 (2.036)	1.00	7
									Q2 (17.872)	1.07 (0.67 ~ 1.71)	
									Q3 (45.473)	1.15 (0.72 ~ 1.83)	
									Q4 (73.073)	0.66 (0.40 ~ 1.10)	
6	Zhang et al. [31]	2019	China	Asian	1508	Case-control	First trimester	2014 Guideline of GDM	< 3.5 h/week (7.875)	1.00	7
									≥3.5 h/week (23.625)	0.739 (0.553 ~ 0.989)	
7	Atlaw et al. [32]	2022	Ethiopia	African	432	Cohort	Second trimester	2013 WHO’s criteria	Low (5)	2.43 (1.11 ~ 5.32)	7
									Moderate (25)	1.98 (0.88 ~ 4.47)	
									High (70)	1.00	
8	Badon et al. [18]	2016	America	Caucasian	3198	Cohort	First trimester	ADA	No activity (0)	1.00	7
									Activity tertile 1 (5.55)	0.83 (0.56 ~ 1.22)	
									Activity tertile 2 (17.55)	0.74 (0.49 ~ 1.12)	
									Activity tertile 2 (30.45)	0.53 (0.33 ~ 0.86)	
9	Chasan-taber et al. [33]	2008	America	Multi-ethnic	1006	Cohort	First trimester	ADA	Q1 (0)	1.00	8
									Q2 (15.75)	0.50 (0.10 ~ 1.60)	
									Q3 (21.0)	0.60 (0.20 ~ 1.70)	
									Q4 (23.625)	0.80 (0.20 ~ 1.90)	
							Second trimester		Q1 (0)	1.00	
									Q2 (15.75)	0.60 (0.20 ~ 1.80)	
									Q3 (21.0)	0.30 (0.10 ~ 1.10)	
									Q4 (23.625)	0.40 (0.10 ~ 1.20)	
10	Chasan-taber et al. [10]	2014	America	Multi-ethnic	1241	Cohort	First trimester	ADA	1st quartile (0)	1.00	8
									2nd quartile (15.75)	0.43 (0.14 ~ 1.27)	
									3rd quartile (23.625)	0.92 (0.39 ~ 2.18)	
									4th quartile (46.0)	0.69 (0.27 ~ 1.73)	
							Second trimester		1st quartile (0)	1.00	
									2nd quartile (15.75)	1.83 (0.59 ~ 5.67)	
									3rd quartile (23.625)	1.26 (0.39 ~ 4.06)	
									4th quartile (46.0)	1.24 (0.38 ~ 4.05)	
11	Dempsey et al. [34]	2004	America	Caucasian	541	Case-control	Second trimester	NDDG	None (0)	1.00	7
									0.1 ~ 1.9 h/week (4.5)	0.96 (0.56 ~ 1.66)	
									2.0 ~ 3.9 h/week (13.275)	0.51 (0.29 ~ 0.90)	
									4.0 ~ 5.9 h/week (22.275)	0.52 (0.25 ~ 1.10)	
									≥6.0 h/week (31.275)	0.53 (0.29 ~ 0.94)	
12	Dempsey et al. [35]	2004	America	Caucasian	909	Cohort	Second trimester	NDDG	None (0)	1.00	7
									< 21.1 MET-h/week (10.55)	0.57 (0.27 ~ 1.21)	
									≥ 21.1 MET-h/week (31.65)	0.26 (0.10 ~ 0.65)	
13	do Nascimento et al. [36]	2017	Brazil	Multi-ethnic	544	Cohort	First trimester	IADPSG	Inactive (7.875)	1.9 (1.1 ~ 3.0)	8
									Active (32.813)	1.0	
14	Dye et al. [37]	1997	America	Caucasian	12,799	Case-control	First trimester	NA	0 time/week (0)	1.00 (0.80 ~ 1.30)	6
									1 ~ 7 times/week(10.125)	1.00	
15	lotfi et al. [38]	2019	Iran	Asian	341	Case-control	Second trimester	NDDG	Low (5)	1.00	7
									Moderate (25)	0.90 (0.60 ~ 1.50)	
									High (70)	0.90 (0.30 ~ 2.80)	
16	Mishra et al. [39]	2018	India	Asian	373	Case-control	Second trimester	Carpenter and Coustan criteria	low-to-Moderate (24.991)	5.90 (3.60 ~ 9.80)	7
									High (74.991)	1.00	
17	Nasiri-Amiri et al. [40]	2016	Iran	Asian	200	Case-control	First trimester	Carpenter and Coustan criteria	Low (15.75)	1.09 (0.30 ~ 3.96)	7
									High (23.625)	1.00	
18	Nguyen et al. [17]	2018	Vietnam	Asian	1987	Cohort	First trimester	2013 WHO’s criteria	1st tertile (33.1)	1.00	8
									2nd tertile (123.2)	0.80 (0.61 ~ 1.05)	
									3rd tertile (237.2)	0.70 (0.53 ~ 0.94)	
19	Oken et al. [41]	2006	America	Caucasian	1805	Cohort	First trimester	ADA	2 or Less h/week (4.5)	1.00	7
									3 ~ 6 h/week (20.25)	0.72 (0.37 ~ 1.40)	
									7 ~ 13 h/week (45.0)	0.59 (0.28 ~ 1.23)	
									14 or more (76.5)	0.91 (0.37 ~ 2.21)	
20	Padmapriya et al. [42]	2017	Singapore	Asian	1083	Cohort	Second trimester	1999 WHO’s criteria	Insufficiently active (5)	1.00	8
									Sufficiently active (25)	0.82 (0.56 ~ 1.20)	
									Highly active (70)	0.56 (0.32 ~ 0.98)	

*ADA* American Diabetes Association, CIs confidence intervals, *GDM* gestational diabetes mellitus, *IADPSG* International Association of the Diabetes and Pregnancy Study Groups, *MET* metabolic equivalent of task, *NA* Not available, *NDDG*, National Diabetes Data Group, *NOS* Newcastle-Ottawa Scale, *ORs* odds ratios, *PA* physical activity, *Q1* 1st quartile, *Q2* 2nd quartile, *Q3* 3rd quartile, *Q4* 4th quartile, *RRs* relative risks, *WHO* World Health Organization

### High versus low PA during pregnancy: analysis and subgroup analyses

**Fig. 2:**
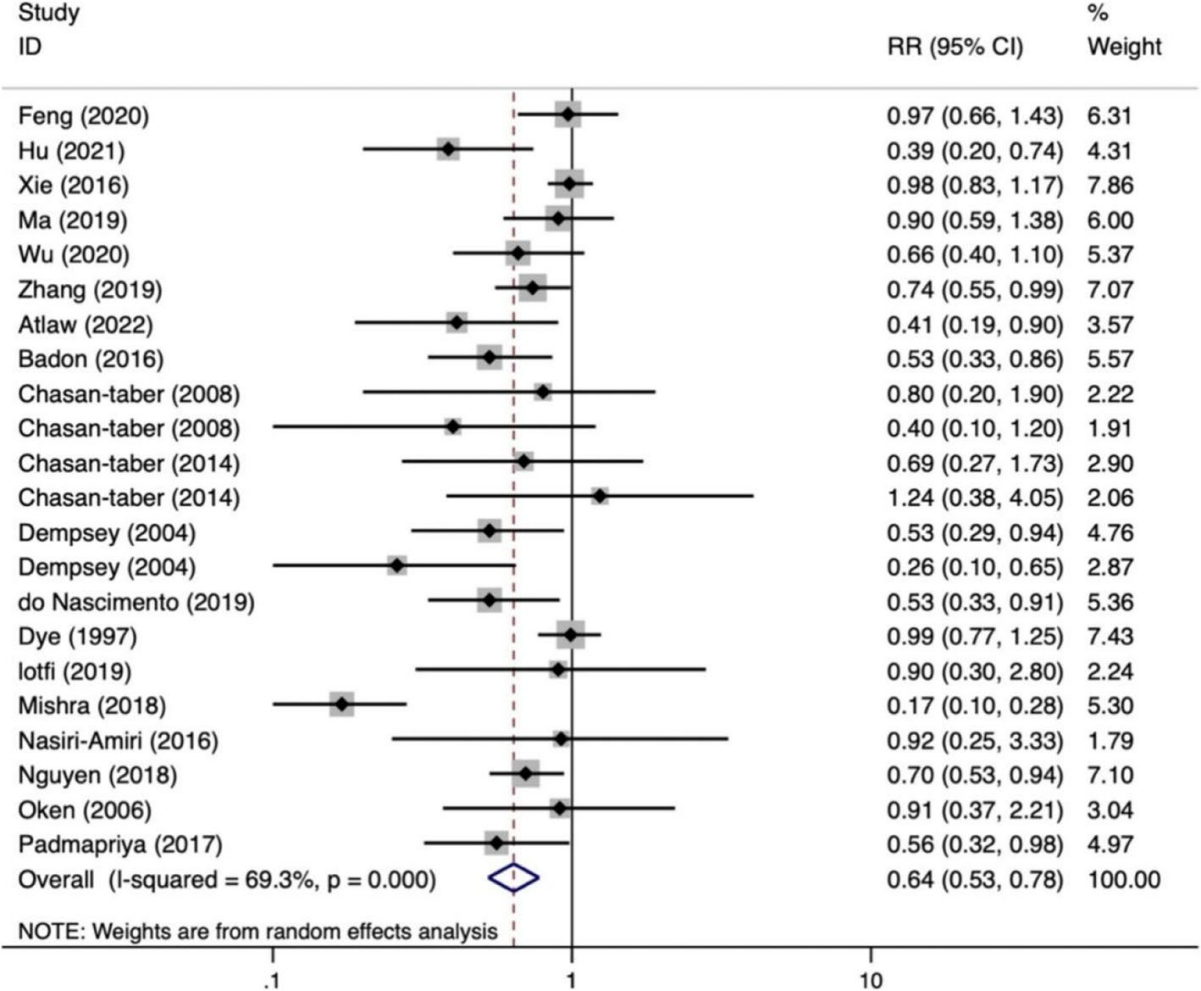
Risk of GDM with PA during pregnancy (high/low)

**Fig. 3:**
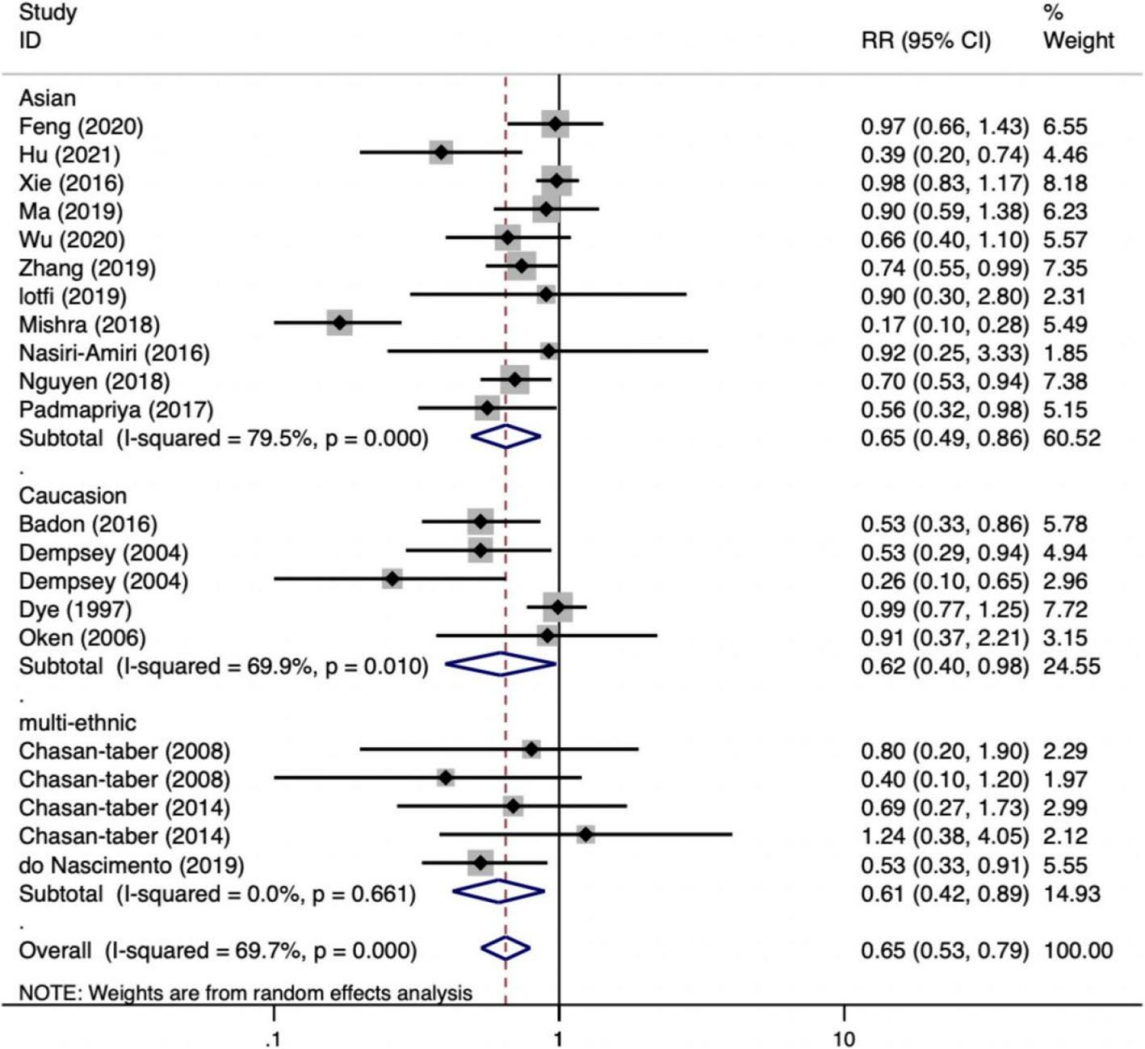
Subgroup analyses for race

According to a meta-analysis of the 22 included studies, the lowest level of PA during pregnancy was linked to a higher risk of GDM (RR = 0.64, 95%CI: 0.53 ~ 0.78; *P* < 0.001). Race subgroup analyses were conducted due to the significant heterogeneity of the studies (*I*^*2*^ = 69.3%). According to one study of an African population, higher levels of PA in the second trimester of pregnancy were associated with a 59% lower risk of GDM (RR = 0.41, 95% CI: 0.19 ~ 0.90). As there was only one African study [32], it was excluded from the subgroup analysis, thereby resulting in the inclusion of 21 studies. The results of the subgroup analysis revealed that heterogeneity appeared to be lower in multi-ethnic populations (*I*^*2*^ = 0.0%). Among Asian and Caucasian populations, higher levels of physical activity during pregnancy were demonstrated to lower the risk of GDM by 35 and 38%, respectively. Similarly, a significant correlation was found in multi-ethnic populations. Specific results are presented in Figs. 2 and 3.

**Fig. 4:**
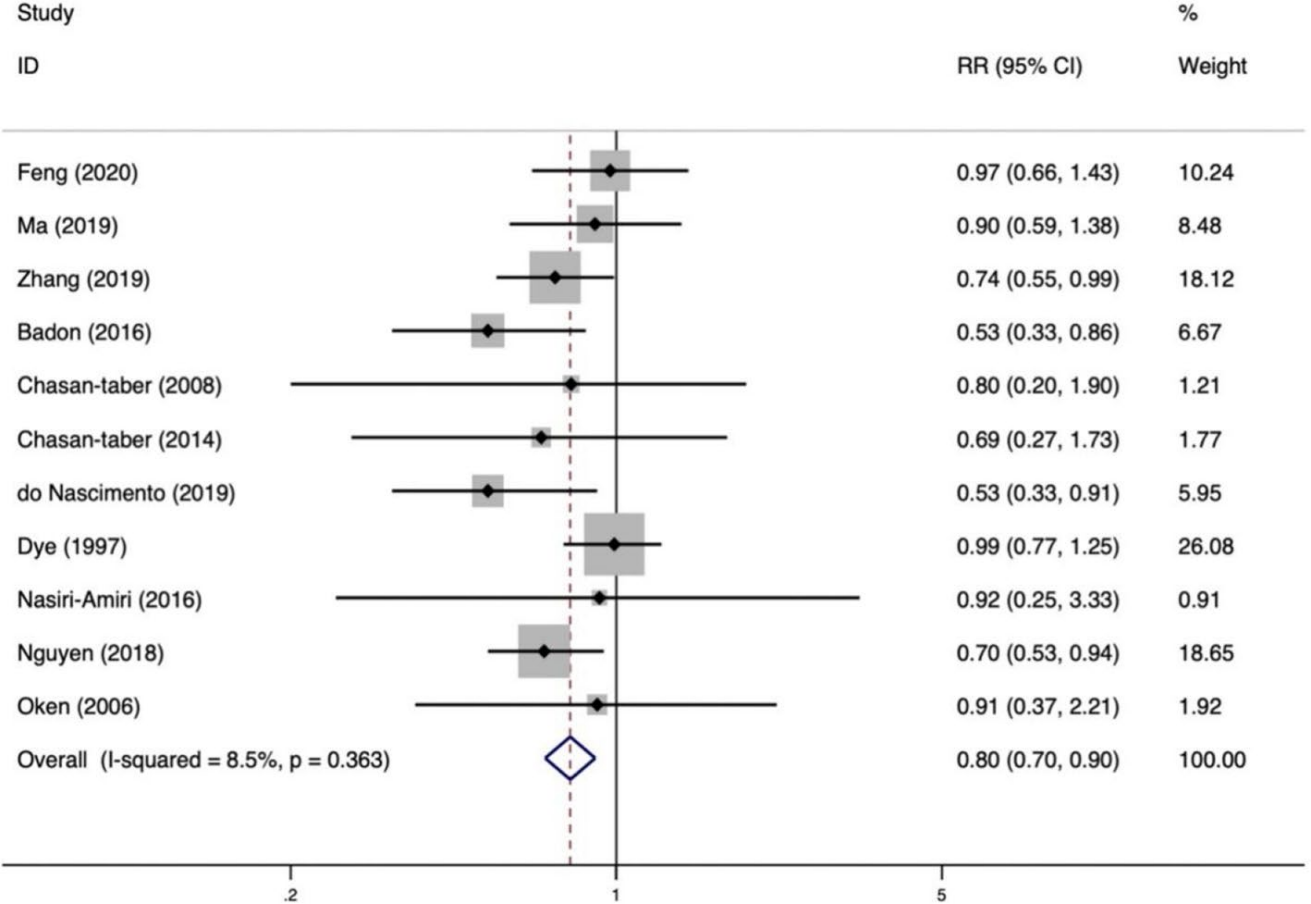
Risk of GDM with PA during the first trimester

**Fig. 5:**
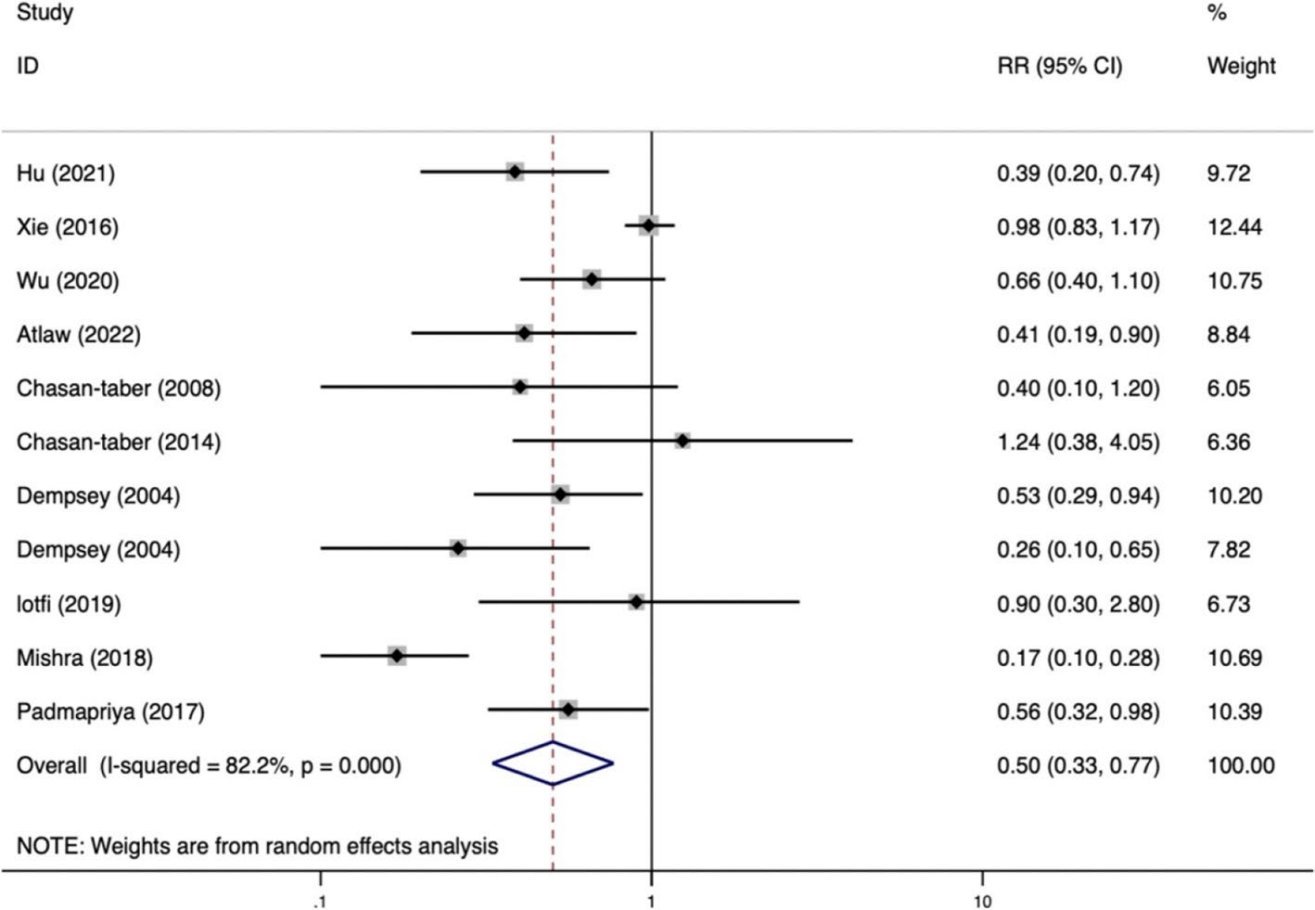
Risk of GDM with PA during the second trimester

We explored the association between PA and the risk of GDM during both first and second trimesters. Eleven studies investigating PA in the first trimester of pregnancy [10, 16–18, 29, 31, 33, 36, 37, 40, 41] as well as 11 studies assessing PA and the risk of GDM in the second trimester [10, 14, 28, 30, 32–35, 38, 39, 42], were included in this review. According to the meta-analysis, a higher level of PA in the first trimester reduced the risk of GDM by 20% (RR = 0.80, 95%CI: 0.70 ~ 0.90), with low heterogeneity between studies (*I*^*2*^ = 8.4%); there was a significant positive correlation between PA during the second trimester and risk of GDM (RR = 0.50, 95%CI: 0.33 ~ 0.77). Figures 4 and 5 are showed the detailed results.

### Dose-response association between PA during pregnancy and incident GDM

Six studies reported PA as a dichotomous variable [29, 31, 36, 37, 39, 40]. As this did not meet the requirement of dose–response analysis, this analysis ultimately included 16 studies [10, 14, 16–18, 28, 30, 32–35, 38, 41, 42].

**Fig. 6:**
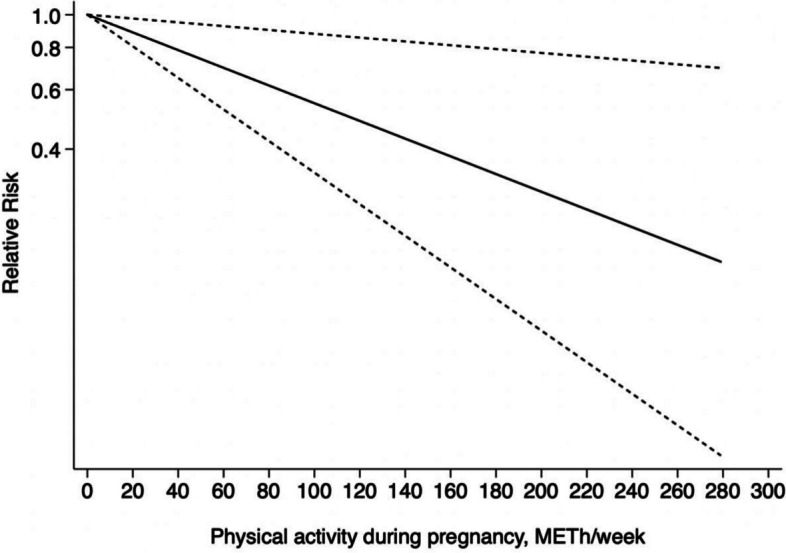
Linear dose–response association between PA during pregnancy and incident GDM

We noticed no evidence of a non-linear relationship between PA during pregnancy and GDM risk (χ^2^ = 3.69, *P*_*nonlinearity*_ *= 0.055* > 0.05) and further discovered that the dose–response relationship between PA and GDM was linear. The risk of GDM was found to be reduced by 6% (RR = 0.94, 95%CI: 0.89 ~ 0.99) among pregnant women who met the 10 MET-h/week level (guidelines recommended minimum PA levels of 150 minutes per week as compared to sedentary individuals). The risk of GDM was further decreased if PA during pregnancy was increased by 20 MET-h/week or even 50 MET-h/week (RR = 0.89, 95%CI: 0.81 ~ 0.97; RR = 0.85, 95%CI: 0.76 ~ 0.94). Figure 6 details the dose–response linear association between pregnancy-related PA and the risk of GDM.

**Fig. 7:**
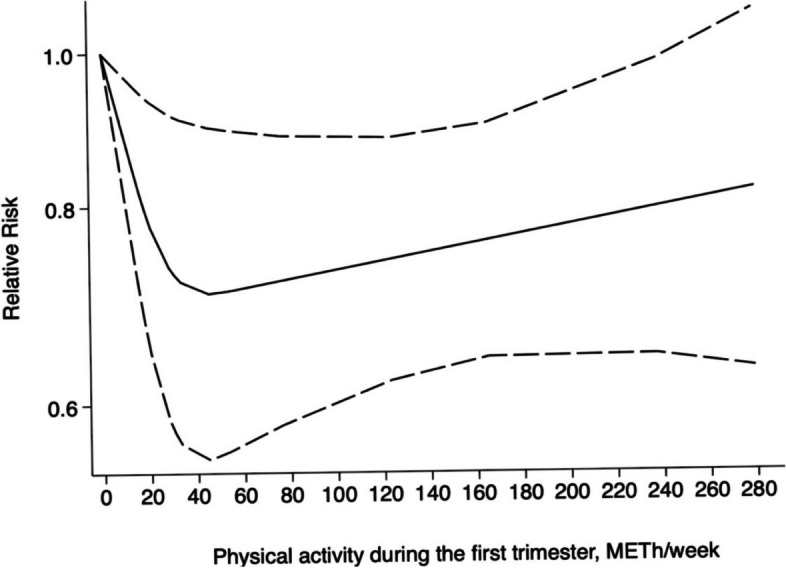
Non-linear association between PA during the first trimester and incident GDM

Additionally, we investigated the dose–response relationship between PA and the risk of GDM in the first and second trimesters of pregnancy, respectively. Six studies were included in the dose–response relationship of PA with GDM during the first trimester; five studies were excluded because they reported PA as a dichotomous variable. A significant roughly L-shaped curvilinear dose–response relationship was observed between PA during the first trimester and incident GDM (χ^2^ = 6.27, *P*_*nonlinearity*_ *= 0.012*). According to these results, any level of PA in the first trimester of pregnancy was associated with a lower risk of GDM. Compared to inactive individuals, results from the cubic spline model suggest that pregnant women who achieved 10 MET-h/week, 20 MET-h/week, and 50 MET-h/week of PA energy expenditure in the first trimester of pregnancy had a 13% (RR = 0.87, 95%CI: 0.79 ~ 0.96), 22% (RR = 0.78, 95%CI: 0.65 ~ 0.93), and 29% lower incidence of GDM (RR = 0.71, 95%CI: 0.55 ~ 0.89), respectively. According to the trend displayed in Fig. 7, the risk of GDM reached the lowest point at a PA level of 50 MET-h/week.

**Fig. 8:**
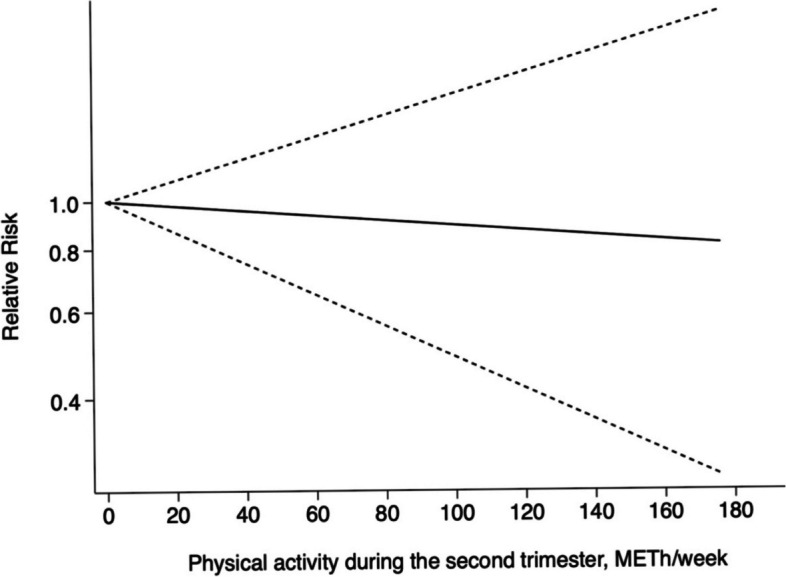
Linear association between PA during the second trimester and incident GDM

We excluded one study that reported PA as a dichotomous variable as it failed to meet the non-linear dose–response analysis criteria, thereby resulting in data from 10 studies being included in the dose–response analysis of PA during the second trimester. Further, we found a linear association between GDM and PA during the second trimester (χ^2^ = 1.19, *P*_*nonlinearity*_ *= 0.276*). The linear analysis indicated that when energy expenditure from PA increased in the second trimester, the risk of GDM tended to decline. Further, the results from the cubic spline model revealed women in the second trimester with a PA of 10 MET-h/week have a 1% lower risk of developing GDM (RR = 0.99, 95%CI: 0.93 ~ 1.05). Figures 7 and 8 illustrate how PA during the first and second trimesters of pregnancy affects the risk of developing GDM.

### Publication Bias, sensitivity analyses and certainty assessment results

**Fig. 9:**
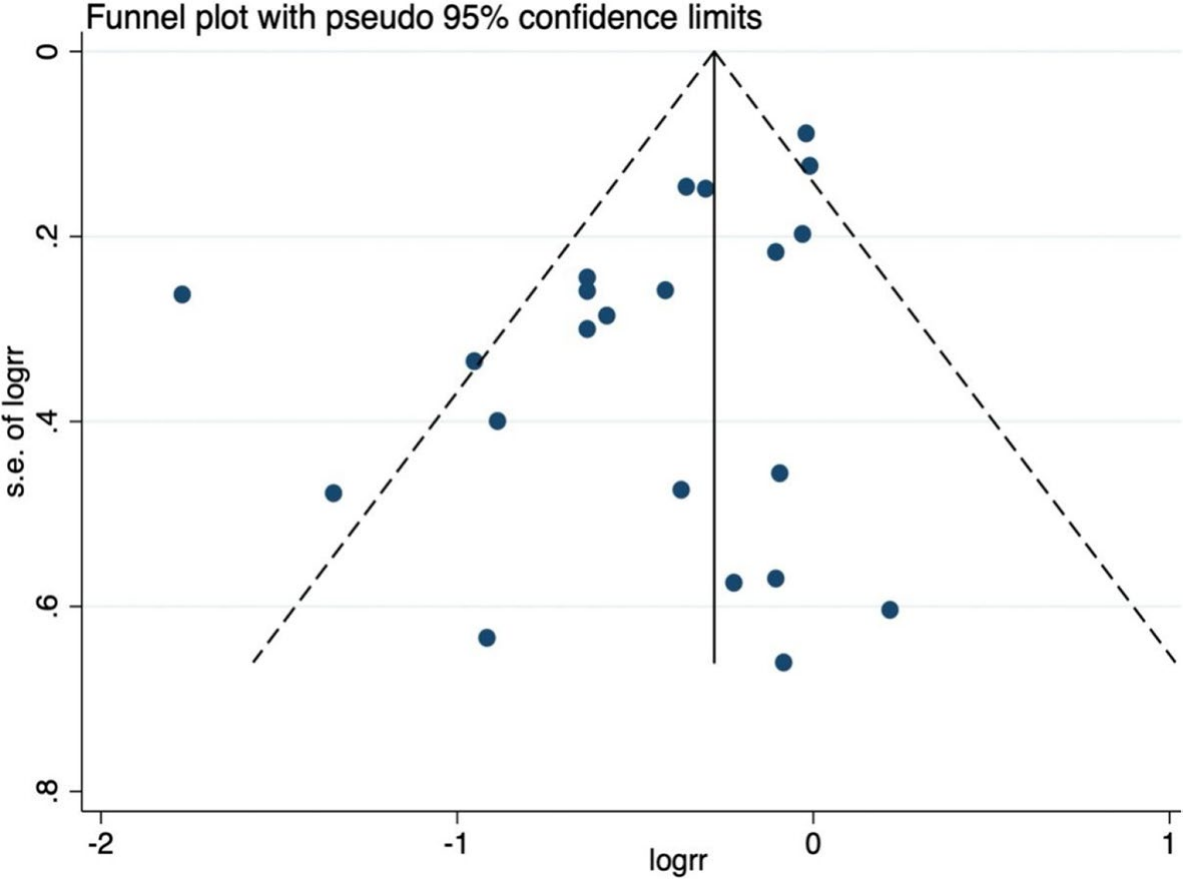
Funnel plots

**Fig. 10:**
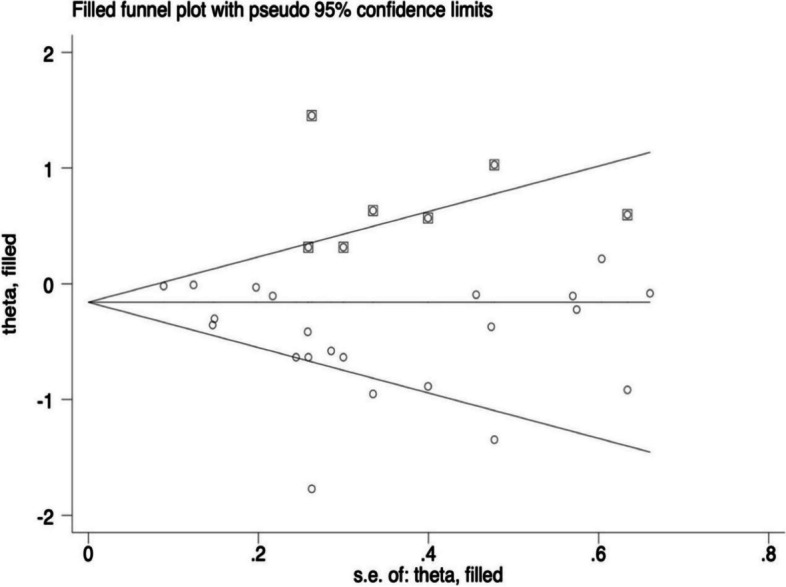
Funnel plots were performed by ‘trim-and-fill’ analysis

Begg’s test indicated no obvious publication bias among the studies (*P = 0.367*), but the results of Egger’s test and the left-to-right asymmetry of the funnel plot did reveal the possible existence of some publication bias among the studies (*P = 0.023*). The trim-and-fill analysis, which uses simple symmetry assumptions and an iterative approach to estimate the number of missing studies, revealed a tiny pre- and post-combined effect size change, thereby indicating a small publication bias and more stable results. The results are presented in Figs. 9 and 10.

**Fig. 11:**
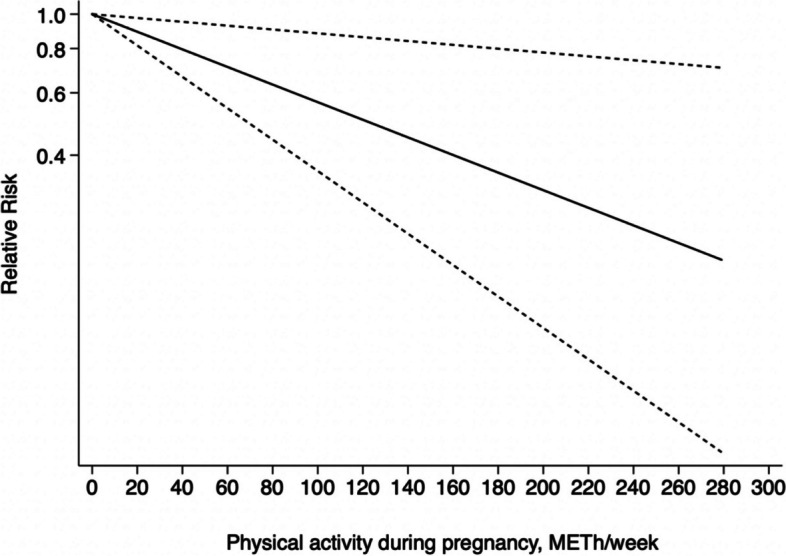
Dose–response association between PA during pregnancy and GDM modelled by using restricted cubic splines applied as 30 min/session

The method of excluding studies one by one and comparing the difference with the combined effect size was used to perform sensitivity analysis, which revealed that the risk was not significantly altered by any of the individual studies. Moreover, the sensitivity analysis involved performing the cubic spline model with a 30-minute time assumption, and the main research dose–response curve remained unchanged. Table 2 and Fig. 11 present the detailed results.

**Table 2:** The results of cubic spline model with a 30-minute time assumption

PA dose during pregnancy (MET-h/week)	*RR*	*95%CI*
10	*0.94*	*(0.90–0.99)*
20	*0.89*	*(0.82–0.98)*
50	*0.85*	*(0.76–0.94)*

*CI* confidence intervals, *MET* metabolic equivalent of task, *PA* physical activity, *RR* relative risk

The result of the certainty assessment for this meta-analysis outcome was convincing (strong evidence). Specific certainty assessment progress is presented in Table S3 in the Supplementary Information.

## Discussion

According to the study’s findings, lower PA during pregnancy increases the risk of GDM. Higher levels of PA during pregnancy were observed to reduce the risk of developing GDM by 36% compared to the lower PA group. This is similar to the conclusions of the meta-analyses by Tobias et al. and Mijatovic-Vukas et al., both of which demonstrated a link between PA during pregnancy and the risk of developing GDM [8, 43]. We additionally investigated the relationship between PA throughout various stages of pregnancy and GDM risk. Consistent with the results of Tobias et al.’s 2011 meta-analysis, we incorporated new data from 2012 to 2023 and combined it with that of Tobias et al.’s study and discovered an inverse relationship between PA during the first trimester of pregnancy and the risk of developing GDM. Moreover, the results demonstrated that increasing PA in the second trimester could decrease the probability of developing GDM; however, the slope of the association in the second trimester was much lower than that in the first trimester. This inconsistency may be attributed to the fact that increased PA in the latter stage may restrict the extent to which the risk of GDM decreases because the second trimester is closer when GDM is diagnosed. In accordance with a randomised controlled trial study, a second-trimester prenatal exercise intervention did not significantly lower the incidence of GDM [44]. In addition, owing to high heterogeneity between the studies and for the variations in effect values among studies, we performed a subgroup analysis by race. Despite the genetic heterogeneity and varying lifestyles among populations, we observed positive results for all races, which indicated that increasing PA during pregnancy could lower the risk of developing GDM in all race groups.

Further, compared to the previous meta-analysis, we concentrated on exploring the dose–response relationship between PA during pregnancy and the risk of GDM. This is the first meta-analysis to quantify PA during the first and second trimesters of pregnancy to examine the dose–response relationship between PA level and incident GDM. We discovered a linear relationship between PA during pregnancy and the risk of developing GDM among pregnant women who engaged in PA amounting to 10 MET-h/week, 20 MET-h/week, and 50 MET-h/week and found that the risk of GDM was decreased 6, 11, and 15%, respectively. We also discovered dose–response correlations between PA and incident GDM in different gestational trimesters. In accordance with Aune et al.’s results [20], our meta-analyses have also revealed evidence of a non-linear association between PA in the first trimester of pregnancy and incident GDM. The risk of GDM decreased continuously as PA increased until the trend stabilized, and the L-shaped dose–response curve became more apparent with the addition of new literature data. In addition, there was a linear dose–response relationship between second trimester PA and GDM risk, with 1, 4 and 15% reductions in GDM risk for increases of 10 MET-h/week, 20 MET-h/week and 50 MET-h/week in PA in the second trimester, respectively. These findings reveal that increasing and maintaining the appropriate amount of PA is critical for preventing and treating GDM.

Extant research also provides evidence on the dose–response relationship between PA and diabetes [45]. By using a pedometer to record the steps of 7118 study subjects, Kraus et al. discovered a linear correlation between daily steps and the onset of T2DM. Increasing the average daily step count from 2000 to 10,000 steps reduced diabetes risk by 5.5%; after adjusting for confounders, the results revealed a total reduction of greater than 6% [46]. Aune et al. and Smith et al. found that higher levels of leisure-time PA in the adult population were associated with a significantly lower incidence of T2DM, and the dose–response relationship between leisure-time PA and T2DM was curvilinear [15, 47]. Similarly, a meta-analysis by Patterson et al. revealed a linear positive connection between sedentary behaviour and T2DM [48]. A previous study explored the dose–response relationship between PA during pregnancy and GDM risk: Hu et al. investigated the dose–response relationship between PA patterns and risks of GDM in 669 pregnant women and discovered a curvilinear link between total physical activity (TPA) in the second trimester, low-intensity PA, and the risk of developing GDM [14]. The research also assessed the energy expenditure thresholds for TPA and low intensity PA, which were 79.800 MET-h/week and 56.575 MET-h/week, respectively, in connection with the risk of GDM. However, the research focused only on a small and monoethnic Chinese population, while our meta-analysis included high-quality original studies with large sample sizes and different ethnicities. Furthermore, we harmonized PA during pregnancy and modelled with restricted cubic spline to determine the dose–response relationship, thereby providing further evidence to confirm the connection between PA during pregnancy and incident GDM.

Diabetes mellitus is a metabolic disease with a complex pathogenesis, and PA or exercise may further reduce the risk of developing GDM by improving the variety of complex mechanisms that cause diabetes. First, the researchers confirmed that regular exercise and long-term PA can increase GLUT-4 content and glycogen synthase activity, thereby improving insulin sensitivity in muscles and other tissues, which strengthens the body by utilising glucose and lowering insulin resistance [49, 50]. In a study conducted at 28 weeks of gestation, Ong et al. revealed that, compared to the control group, the PA-based intervention group had lower 1 h (*P* = 0.07) and 2 h (*P* = 0.08) glucose levels during the oral glucose tolerance test (OGTT) [51]. Similarly, a systematic review that analysed the benefits of PA has in controlling GDM also reported that resistance or aerobic exercise is effective for the control of insulin [52]. Second, as β-cell dysfunction is one of the causes of diabetes, increasing PA has been shown to lower the incidence of diabetes by enhancing β-cell function. Moderate PA can protect β-cell function and slow the development of diabetes by promoting β-cell proliferation as well as reducing oxidative stress and inflammatory response [53, 54]. Nieuwoudt et al. found that pancreatic β-cell function was significantly enhanced and insulin secretion improved after functional high-intensity exercise training in adults with T2DM [55]. In addition, one of the independent risk factors for GDM is overweight and obesity [56], and short-term weight gain during pregnancy increases the risk of developing GDM. In this case, promoting PA or exercise during pregnancy is an effective strategy for controlling weight to prevent GDM. According to a review, the use of resistance exercise-only interventions, whether in healthy or obese adults, effectively reduced visceral fat and controlled blood glucose [57]. A cohort study from China also indicated that weight gain and a higher BMI during pregnancy tend to increase the risk of GDM; thus, PA during pregnancy plays a significant role in maintaining energy balance and weight control, which lowers the risk of diabetes [58].

GDM is of great concern in the field of public health, the phenomenon of ‘paying attention to treatment and neglecting prevention’ is typically noted in GDM clinical studies [59]. Considering gestation is a special period for women, the clinical application of medical therapy is restricted to a certain extent. Thus, both the medical and sports science fields recognize that increasing PA levels during pregnancy is an efficient strategy to address this public health issue [60, 61]. In fact, the levels of PA during pregnancy worldwide remain generally inadequate. According to a Brazilian study that used accelerometers to assess PA during pregnancy, 2317 pregnant women averaged only 14 minutes of MVPA per day [62]. Similarly, in a study of pregnant women from the United States via objective measures of PA during pregnancy, it was discovered that these women engaged in an average of merely 11.5 minutes/day of PA during the first trimester [63]. The majority of Asian pregnant women are influenced by traditional opinions that PA levels during gestation must be limited. Yin et al. used a validated questionnaire to investigate 201 pregnant women in Singapore regarding their daily PA levels; they discovered that only 12.6% of the participants exercised for at least 150 minutes a week, as recommended by guidelines [64]. Further, women at 24 hospitals across 15 provinces in China were reported approximately 68.5% failure to participate in sufficient PA [65]. Hence, it is vital to explore appropriate strategies to encourage inactive women to increase their PA levels during pregnancy.

A common approach adopted by nations for solving the issue of insufficient pregnancy-related PA is the establishment of PA guidelines. In 1985, the ACOG released the first guidelines regarding PA during pregnancy, which indicated that aerobic exercise is beneficial for pregnant women [66]. To guarantee safety of pregnancy, ACOG updated its guidelines in 1994, 2002, 2015, and 2020 to clarify the specific intensity of PA during pregnancy; the latest guideline recommended that MVPA should be engaged in for at least 20–30 minutes per day on most or all days of the week [12, 67–69]. Similarly, the U.S. Department of Health and Human Service (USDHHS) established the 2008 Physical Activity Guidelines for Americans and released its second edition in 2018 to emphasize that women during pregnancy should engage in at least 150 minutes of moderate-intensity aerobic exercise per week [70, 71]. In addition, guidelines on PA during pregnancy were also released by the UK and Canada, offering similar suggestions on exercise during pregnancy [72, 73]. However, the recommendations from current pregnancy-related PA guidelines are similar to the WHO guideline for PA in the general population [74]. Considering the specificity of gestation for each woman, it is essential to provide pregnancy-specific PA suggestions in future guidelines. Our systematic review revealed the dose–response relationship between the PA during pregnancy and the risk of developing GDM, which could provide a scientific basis for developing optimal PA guidelines for pregnancy as well as more effective public health policies in this regard.

### Limitations

Our meta-analysis has a few limitations: (1) As all of the included studies used questionnaires to investigate PA during pregnancy, there is the inevitable weakness of recall bias; (2) smoking, diet, and body mass index (BMI) were all confounding variables that influenced the study’s findings. While most included research studies corrected their results for these confounding variables by providing results for unadjusted covariates, the meta-analysis results of our study may be affected by these confounding factors; (3) while we quantified PA using MET-h/week as a unified unit to calculate the dose, other studies classified PA differently, and the parameters for frequency, intensity, and duration varied widely, probably influencing the accuracy of our results.

## Conclusion

To summarize, we found a dose–response relationship between PA during pregnancy and incident GDM—increasing PA during pregnancy has a positive effect on reducing the risk of GDM. Our results indicated a significant non-linear dose–response in the first trimester and a linear relationship in the second trimester. An adequate increase in PA in the first and second trimesters of pregnancy could prevent GDM, with increased PA during the first trimester being particularly effective.

In order to further promote the health of pregnant women, our review recommends that both the guidelines for PA during pregnancy and public health policies must encourage pregnant women to increase their PA levels in the first and second trimesters—particularly to achieve a PA level of 50 MET-h/week in the first trimester that will produce the optimum GDM prevention effects.

### Supplementary Information

**Supplementary Material 1.**

## Data Availability

All data are available from the corresponding author on reasonable request: Liuwei Zhang (2466@bsu.edu.cn) / Jiaoying Cheng (cjy19781031@163.com).
