# Supplementary material for: Physical activity during pregnancy and the risk of gestational diabetes mellitus: a systematic review and dose–response meta-analysis
Source: BMC Public Health. 2024 Feb 23;24:594. doi: 10.1186/s12889-024-18131-7 (PMC10893683; doi:10.1186/s12889-024-18131-7)
Supplement: Supplementary file 1 — Supplementary Material 1. [file 12889_2024_18131_MOESM1_ESM.docx]

**Physical activity during pregnancy and the risk of gestational diabetes mellitus: A systematic review and dose-response meta-analysis**

**Supplementary Information**

**PRISMA 2020 checklist**

| **Section and Topic** | **Item #** | **Checklist item** | **Location where item is reported** |
| --- | --- | --- | --- |
| **TITLE** | | |  |
| Title | 1 | Identify the report as a systematic review. | **Page1** |
| **ABSTRACT** | | |  |
| Abstract | 2 | See the PRISMA 2020 for Abstracts checklist. | **Pages 2-3** |
| **INTRODUCTION** | | |  |
| Rationale | 3 | Describe the rationale for the review in the context of existing knowledge. | **Pages 3-6** |
| Objectives | 4 | Provide an explicit statement of the objective(s) or question(s) the review addresses. | **Pages 5-6** |
| **METHODS** | | |  |
| Eligibility criteria | 5 | Specify the inclusion and exclusion criteria for the review and how studies were grouped for the syntheses. | **Pages 6-7** |
| Information sources | 6 | Specify all databases, registers, websites, organisations, reference lists and other sources searched or consulted to identify studies. Specify the date when each source was last searched or consulted. | **Pages 6-7** |
| Search strategy | 7 | Present the full search strategies for all databases, registers and websites, including any filters and limits used. | **Pages 6-7, Table S1** |
| Selection process | 8 | Specify the methods used to decide whether a study met the inclusion criteria of the review, including how many reviewers screened each record and each report retrieved, whether they worked independently, and if applicable, details of automation tools used in the process. | **Pages 6-7** |
| Data collection process | 9 | Specify the methods used to collect data from reports, including how many reviewers collected data from each report, whether they worked independently, any processes for obtaining or confirming data from study investigators, and if applicable, details of automation tools used in the process. | **Pages 7-8** |
| Data items | 10a | List and define all outcomes for which data were sought. Specify whether all results that were compatible with each outcome domain in each study were sought (e.g. for all measures, time points, analyses), and if not, the methods used to decide which results to collect. | **Pages 7-8** |
|  | 10b | List and define all other variables for which data were sought (e.g. participant and intervention characteristics, funding sources). Describe any assumptions made about any missing or unclear information. | **Pages 7-8** |
| Study risk of bias assessment | 11 | Specify the methods used to assess risk of bias in the included studies, including details of the tool(s) used, how many reviewers assessed each study and whether they worked independently, and if applicable, details of automation tools used in the process. | **Page8** |
| Effect measures | 12 | Specify for each outcome the effect measure(s) (e.g. risk ratio, mean difference) used in the synthesis or presentation of results. | **Pages 8-9** |
| Synthesis methods | 13a | Describe the processes used to decide which studies were eligible for each synthesis (e.g. tabulating the study intervention characteristics and comparing against the planned groups for each synthesis (item #5)). | **Pages 8-9** |
|  | 13b | Describe any methods required to prepare the data for presentation or synthesis, such as handling of missing summary statistics, or data conversions. | **Pages 8-9** |
|  | 13c | Describe any methods used to tabulate or visually display results of individual studies and syntheses. | **Pages 8-9** |
|  | 13d | Describe any methods used to synthesize results and provide a rationale for the choice(s). If meta-analysis was performed, describe the model(s), method(s) to identify the presence and extent of statistical heterogeneity, and software package(s) used. | **Pages 8-9** |
|  | 13e | Describe any methods used to explore possible causes of heterogeneity among study results (e.g. subgroup analysis, meta-regression). | **Pages 8-9** |
|  | 13f | Describe any sensitivity analyses conducted to assess robustness of the synthesized results. | **Pages 8-9** |
| Reporting bias assessment | 14 | Describe any methods used to assess risk of bias due to missing results in a synthesis (arising from reporting biases). | **Pages 8-9** |
| Certainty assessment | 15 | Describe any methods used to assess certainty (or confidence) in the body of evidence for an outcome. | **Page9** |
| **RESULTS** | | |  |
| Study selection | 16a | Describe the results of the search and selection process, from the number of records identified in the search to the number of studies included in the review, ideally using a flow diagram. | **Page10, Figure.1** |
|  | 16b | Cite studies that might appear to meet the inclusion criteria, but which were excluded, and explain why they were excluded. | **Page10, Figure.1** |
| Study characteristics | 17 | Cite each included study and present its characteristics. | **Page10, Table 1.** |
| Risk of bias in studies | 18 | Present assessments of risk of bias for each included study. | **Table 1.** |
| Results of individual studies | 19 | For all outcomes, present, for each study: (a) summary statistics for each group (where appropriate) and (b) an effect estimate and its precision (e.g. confidence/credible interval), ideally using structured tables or plots. | **Figures 2-8.** |
| Results of syntheses | 20a | For each synthesis, briefly summarise the characteristics and risk of bias among contributing studies. | **Figures 2-8.** |
|  | 20b | Present results of all statistical syntheses conducted. If meta-analysis was done, present for each the summary estimate and its precision (e.g. confidence/credible interval) and measures of statistical heterogeneity. If comparing groups, describe the direction of the effect. | **Figures 2-8.** |
|  | 20c | Present results of all investigations of possible causes of heterogeneity among study results. | **Figure 3** |
|  | 20d | Present results of all sensitivity analyses conducted to assess the robustness of the synthesized results. | **Table 2, Figure 11** |
| Reporting biases | 21 | Present assessments of risk of bias due to missing results (arising from reporting biases) for each synthesis assessed. | **Figures 9,10.** |
| Certainty of evidence | 22 | Present assessments of certainty (or confidence) in the body of evidence for each outcome assessed. | **Page26, TableS3** |
| **DISCUSSION** | | |  |
| Discussion | 23a | Provide a general interpretation of the results in the context of other evidence. | **Pages 26-31** |
|  | 23b | Discuss any limitations of the evidence included in the review. | **Page31** |
|  | 23c | Discuss any limitations of the review processes used. | **Page31** |
|  | 23d | Discuss implications of the results for practice, policy, and future research. | **Page31** |
| **OTHER INFORMATION** | | |  |
| Registration and protocol | 24a | Provide registration information for the review, including register name and registration number, or state that the review was not registered. | **Page6** |
|  | 24b | Indicate where the review protocol can be accessed, or state that a protocol was not prepared. | **/** |
|  | 24c | Describe and explain any amendments to information provided at registration or in the protocol. | **/** |
| Support | 25 | Describe sources of financial or non-financial support for the review, and the role of the funders or sponsors in the review. | **Page33** |
| Competing interests | 26 | Declare any competing interests of review authors. | **Page32** |
| Availability of data, code and other materials | 27 | Report which of the following are publicly available and where they can be found: template data collection forms; data extracted from included studies; data used for all analyses; analytic code; any other materials used in the review. | **/** |

**Table S1. Systematic literature review search terms and strategies**

| **Search terms for PubMed** |
| --- |
| #1 (pregnancy[Title/Abstract] OR pregnant women[Title/Abstract] OR maternal[Title/Abstract] OR gestation[Title/Abstract]) |
| #2 (Gestational Diabetes Mellitus[Title/Abstract] OR Blood glucose[Title/Abstract]) |
| #3 (exercise[Title/Abstract] OR physical activity[Title/Abstract] OR physical fitness[Title/Abstract] OR sport[Title/Abstract] OR lifestyle intervention[Title/Abstract] OR exercise intervention[Title/Abstract]) |
| *#1 AND #2 AND #3* |
| **Search terms for EBSCO** |
| #1 SU=(pregnancy OR pregnant women OR maternal OR gestation) |
| #2 SU=(Gestational Diabetes Mellitus OR Blood glucose) |
| #3 SU=(exercise OR physical activity OR physical fitness OR sport OR lifestyle intervention OR exercise intervention) |
| *#1 AND #2 AND #3* |
| **Search terms for Web of Science** |
| #1 TI=(pregnancy or gestation or pregnant women or maternal) |
| #2 TI=(Gestational Diabetes Mellitus or Blood glucose) |
| #3 TI=(exercise or physical activity or physical fitness or sport or lifestyle intervention or exercise intervention) |
| *#1 AND #2 AND #3* |
| **Search terms for CNKI (in Chinese)** |
| #1 (TI=pregnancy+pregnant women+maternal+gestation OR KY=pregnancy+pregnant women+maternal+gestation) |
| #2 (TI=Gestational Diabetes Mellitus+Blood glucose OR KY=Gestational Diabetes Mellitus+Blood glucose) |
| #3 (TI=exercise+physical activity+physical fitness+sport+lifestyle intervention+exercise intervention OR KY=exercise+physical activity+physical fitness+sport+lifestyle intervention+exercise intervention) |
| *#1 AND #2 AND #3* |

**Table S1. (Continued)**

| **Search terms for VIP (in Chinese)** |
| --- |
| #1 (M=pregnancy+pregnant women+maternal+gestation) |
| #2 (M=Gestational Diabetes Mellitus+Blood glucose) |
| #3 (M=exercise+physical activity+physical fitness+sport+lifestyle intervention+exercise intervention) |
| *#1 AND #2 AND #3* |
| **Search terms for Wanfang (in Chinese)** |
| #1 (Title or Keywords: (("pregnancy")or("pregnant women")or("maternal")or("gestation"))) |
| #2 (Title or Keywords: (("Gestational Diabetes Mellitus")or("Blood glucose"))) |
| #3 (Title or Keywords: (("exercise") or ("physical activity")or("physical fitness")or("sport")or("lifestyle intervention")or("exercise intervention"))) |
| *#1 AND #2 AND #3* |

**Table S2. PECOS (Population, Exposure, Comparison, Outcome and Study Design) of each study**

| **Study** | **Population** | **Exposure** | **Comparison** | **Outcome** | **Study Design** |
| --- | --- | --- | --- | --- | --- |
| Feng, 2020 | 909 pregnant women participated in the Chinese Pregnant Women Cohort Study | High and moderate physical activity level during 1st trimester | Low physical activity level during 1st trimester | The overall GDM prevalence of pregnant women and the prevalence of GDM in different physical activity during pregnancy level group | Cohort |
| Hu, 2021 | 669 pregnant women in Guangdong Province | High and moderate physical activity level during 2nd trimester | Low physical activity level during 2nd trimester | The overall GDM prevalence of pregnant women and the prevalence of GDM in different physical activity during pregnancy level group | Case-control |
| Xie, 2016 | 6,211 pregnant women with single birth in the First Affiliated Hospital of Shanxi Medical University． | High and moderate physical activity level during 2nd trimester | Low physical activity level during 2nd trimester | The overall GDM prevalence of pregnant women and the prevalence of GDM in different physical activity during pregnancy level group | Case-control |
| Ma, 2019 | 3,646 pregnant women came to 24 hospitals in 15provices for the first-time antenatal care in the first trimester. | High physical activity level during 1st trimester | None physical activity during 1st trimester | The overall GDM prevalence of pregnant women and the prevalence of GDM in different physical activity during pregnancy level group | Cohort |
| Wu, 2020 | 1,083 pregnant women in Guangdong Province | High and moderate physical activity level during 2nd trimester | Low physical activity level during 2nd trimester | The overall GDM prevalence of pregnant women and the prevalence of GDM in different physical activity during pregnancy level group | Cohort |
| Zhang, 2019 | 1,508 pregnant women in Sichuan Province | High physical activity level during 1st trimester | Low physical activity level during 1st trimester | The overall GDM prevalence of pregnant women and the prevalence of GDM in different physical activity during pregnancy level group | Case-control |
| Atlaw, 2022 | 432 pregnant women with a gestational age of 20 weeks in Southeast Ethiopia | High and moderate physical activity level during 2nd trimester | Low physical activity level during 2nd trimester | The overall GDM prevalence of pregnant women and the prevalence of GDM in different physical activity during pregnancy level group | Cohort |
| Badon, 2016 | 3,198 pregnant women initiating prenatal care at clinics associated with Swedish Medical Center and Tacoma General Hospital in Washington State were recruited from 1996 to 2008 | High and moderate physical activity level during 1st trimester | None physical activity during 1st trimester | The overall GDM prevalence of pregnant women and the prevalence of GDM in different physical activity during pregnancy level group | Cohort |
| Chasan-taber,2008 | 1,006 Hispanic pregnant women aged 16–40 years were recruited from 2000 through 2003 | High and moderate physical activity level during 1st trimester | Low physical activity level during 1st trimester | The overall GDM prevalence of pregnant women and the prevalence of GDM in different physical activity during pregnancy level group | Cohort |
| Chasan-taber,2008 | 1,006 Hispanic pregnant women aged 16–40 years were recruited from 2000 through 2003 | High and moderate physical activity level during 2nd trimester | Low physical activity level during 2nd trimester | The overall GDM prevalence of pregnant women and the prevalence of GDM in different physical activity during pregnancy level group | Cohort |

**Table S2. (Continued)**

| **Study** | **Population** | **Exposure** | **Comparison** | **Outcome** | **Study Design** |
| --- | --- | --- | --- | --- | --- |
| Chasan-taber,  2014 | 1,241 Hispanic women | High and moderate physical activity level during 1st trimester | Low physical activity level during 1st trimester | The overall GDM prevalence of pregnant women and the prevalence of GDM in different physical activity during pregnancy level group | Cohort |
| Chasan-taber,  2014 | 1,241 Hispanic women | High and moderate physical activity level during 2nd trimester | Low physical activity level during 2nd trimester | The overall GDM prevalence of pregnant women and the prevalence of GDM in different physical activity during pregnancy level group | Cohort |
| Dempsey,2004 | 541 pregnant women were recruited from April 1998 through February 2001 as part of a case-control study primarily designed | High and moderate physical activity level during 2nd trimester | Low physical activity level during 2nd trimester | The overall GDM prevalence of pregnant women and the prevalence of GDM in different physical activity during pregnancy level group | Case-control |
| Dempsey,2004 | 909 pregnant women was drawn from participants of the ongoing OMEGA Study | High and moderate physical activity level during 2nd trimester | None physical activity during 2nd trimester | The overall GDM prevalence of pregnant women and the prevalence of GDM in different physical activity during pregnancy level group | Cohort |
| do Nascimento,  2017 | 544 Pregnant women were recruited in Brazil | Active physical activity level during 1st trimester | Inactive physical activity level during 1st trimester | The overall GDM prevalence of pregnant women and the prevalence of GDM in different physical activity during pregnancy level group | Cohort |
| Dye,1997 | 12,799 Women who delivered a livebirth in the 15-county region of central New York State between October 1, 1995, and July 31, 1996. | High and moderate physical activity level during 1st trimester | None physical activity during 1st trimester | The overall GDM prevalence of pregnant women and the prevalence of GDM in different physical activity during pregnancy level group | Case-control |
| lotfi,2019 | 341 pregnant women in Iran | High and moderate physical activity level during 2nd trimester | Low physical activity level during 2nd trimester | The overall GDM prevalence of pregnant women and the prevalence of GDM in different physical activity during pregnancy level group | Case-control |
| Mishra,2018 | 373 pregnant women in India | High physical activity level during 2nd trimester | Low-to-moderate physical activity level during 2nd trimester | The overall GDM prevalence of pregnant women and the prevalence of GDM in different physical activity during pregnancy level group | Case-control |
| Nasiri-Amiri,  2016 | 200 pregnant females were recruited in Iran | High physical activity level during 1st trimester | Low physical activity level during 1st trimester | The overall GDM prevalence of pregnant women and the prevalence of GDM in different physical activity during pregnancy level group | Case-control |

**Table S2. (Continued)**

| **Study** | **Population** | **Exposure** | **Comparison** | **Outcome** | **Study Design** |
| --- | --- | --- | --- | --- | --- |
| Nguyen,  2018 | 1,987 pregnant women in Vietnam | High and moderate physical activity level during 1st trimester | Low physical activity level during 1st trimester | The overall GDM prevalence of pregnant women and the prevalence of GDM in different physical activity during pregnancy level group | Cohort |
| Oken,  2006 | 1,805 pregnant women in eastern Massachusetts | High and moderate physical activity level during 1st trimester | Low physical activity level during 1st trimester | The overall GDM prevalence of pregnant women and the prevalence of GDM in different physical activity during pregnancy level group | Cohort |
| Padmapriya,  2017 | 1,083 pregnant women attending their first-trimester were recruited by Growing Up in Singapore Towards healthy Outcomes (GUSTO) mother-offspring cohort study | Highly and sufficiently active physical activity level during 2nd trimester | Insufficiently active physical activity level during 2nd trimester | The overall GDM prevalence of pregnant women and the prevalence of GDM in different physical activity during pregnancy level group | Cohort |

**Table S3. The Certainty assessment results**

| **levels of conclusion** | **Required Conditions** | **Assessed conclusion**  **in this meta-analysis** |
| --- | --- | --- |
| Convincing  (Strong evidence) | Evidence from more than one study type | **Yes** |
|  | Evidence from at least two independent cohort studies | **Yes** |
|  | No substantial unexplained heterogeneity within or between study type or in different populations relating to the presence or absence of an association, or direction of effect | **Yes** |
|  | Good-quality studies to exclude with confidence the possibility that the observed association results from random or systematic error, including confounding, measurement error and selection bias | **Yes** |
|  | Presence of a plausible biological gradient (‘dose-response’) in the association. Such a gradient need not be linear or even in the same direction across the different levels of exposure, so long as this can be explained plausibly | **Yes** |
|  | Strong and plausible experimental evidence, either from human studies or relevant animal models, that typical human exposures can lead to relevant outcomes | **Yes** |
| Probable  (Strong evidence) | Evidence from at least two independent cohort studies or at least five case-control studies | **/** |
|  | No substantial unexplained heterogeneity between or within study type in the presence or absence of an association, or direction of effect |  |
|  | Good-quality studies to exclude with confidence the possibility that the observed association results from random or systematic error, including confounding, measurement error and selection bias |  |
|  | Evidence for biological plausibility |  |
| Limited-Suggestive | Evidence from at least two independent cohort studies or at least five case-control studies | **/** |
|  | The direction of effect is generally consistent though some unexplained heterogeneity may be present |  |
|  | Evidence for biological plausibility |  |
| Limited-No conclusion | Evidence is so limited that no firm conclusion can be made | **/** |

**Table S3. (Continued)**

| **levels of conclusion** | **Required Conditions** | **Assessed Conclusion**  **in the meta-analysis** |
| --- | --- | --- |
| Substantial effect on risk unlikely | Evidence from more than one study type | **/** |
|  | Evidence from at least two independent cohort studies |  |
|  | Summary estimate of effect close to 1.0 for comparison of high-versus low-exposure categories |  |
|  | No substantial unexplained heterogeneity within or between study type or in different populations |  |
|  | Good-quality studies to exclude with confidence the possibility that the observed association results from random or systematic error, including confounding, measurement error and selection bias |  |
|  | Absence of a demonstrable biological gradient (‘dose-response’) |  |
|  | Absence of strong and plausible experimental evidence, from either human studies or relevant animal models, that typical human exposures can lead to relevant outcomes |  |
| The Certainty assessment results for this meta-analysis: **Convincing (Strong evidence)** | | |
